# Exploring the potential of combining IL-2-activated NK cells with an anti-PDL1 monoclonal antibody to target multiple myeloma-associated macrophages

**DOI:** 10.1007/s00262-022-03365-4

**Published:** 2023-01-19

**Authors:** Femke A. I. Ehlers, Niken M. Mahaweni, Annet van de Waterweg Berends, Thara Saya, Gerard M. J. Bos, Lotte Wieten

**Affiliations:** 1 Department of Transplantation Immunology, Tissue Typing Laboratory, Maastricht University Medical Center+, Maastricht, The Netherlands; 2 Department of Internal Medicine, Division of Hematology, Maastricht University Medical Center+, Maastricht, The Netherlands; 3 GROW School for Oncology and Developmental Biology, Maastricht University, Maastricht, The Netherlands

**Keywords:** NK cells, ADCC, Tumor-associated cells, Tumor microenvironment

## Abstract

Multiple myeloma (MM) is an incurable disease, characterized by malignant plasma cells in the bone marrow. MM growth is largely dependent on the tumor microenvironment (TME), consisting of complex cellular networks that shape a tumor-permissive environment. Within the TME, tumor-associated cells (TAC) comprise heterogeneous cell populations that collectively support immunosuppression. Reshaping the TME toward an immunostimulatory environment may enhance effectiveness of immunotherapies. Here, we investigated interactions between donor-derived natural killer (NK) cells and TAC, like tumor-associated macrophages (TAM) and M1 macrophages, and assessed whether anti-tumor effector functions of NK cells could be enhanced by an ADCC-triggering antibody targeting macrophages. Monocytes were polarized in vitro toward either M1 or TAM before co-culture with high-dose IL-2-activated NK cells. NK cell responses were assessed by measuring degranulation (CD107a) and IFN-γ production. We found that NK cells degranulated and produced IFN-γ upon interaction with both macrophage types. NK cell responses against PD-L1^+^ M1 macrophages could be further enhanced by Avelumab, an anti-PD-L1- and ADCC-inducing antibody. Additionally, NK cell responses were influenced by HLA class I, shown by stronger degranulation in NK cell subsets for which the corresponding HLA ligand was absent on the macrophage target cells (KIR-ligand mismatch) compared to degranulation in the presence of the HLA ligand (KIR-ligand match). Our results suggest that NK cells could, next to killing tumor cells, get activated upon interaction with TAC, like M1 macrophages and TAMs, and that NK cells combined with PD-L1 blocking antibodies with ADCC potential could, through IFN-γ secretion, promote a more immune-favorable TME.

**Supplementary Information:**

The online version contains supplementary material available at 10.1007/s00262-022-03365-4.

## Introduction

Multiple myeloma (MM) is an incurable hematological cancer, characterized by the accumulation of malignant plasma cells in the bone marrow (BM). Although therapeutic agents such as immunomodulatory drugs and monoclonal antibodies have substantially prolonged survival, tumor cells frequently develop resistance to therapy due to mutations and support from the tumor microenvironment (TME) [1].

MM cells are dependent on the BM microenvironment to grow uncontrollably. Interaction with BM stromal cells and osteoclasts can, for example, support MM proliferation and survival through direct cell–cell contacts and soluble factors such as IL-6 [2]. Moreover, the TME of MM patients is characterized by changes in the cellular composition compared to normal BM, already early during disease development [3]. BM-infiltrating cells represent highly heterogeneous cell populations, and together, they contribute to suppression of anti-tumor immune responses through various mechanisms such as secretion of suppressive cytokines, nitric oxide, reactive oxygen species, indoleamine-2,3-dioxygenase (IDO) and prostaglandin E2 [PGE2), as well as lactate produced by MM cells [4, 5]. Moreover, immature myeloid cells, which are often described as myeloid-derived suppressor cells, as well as regulatory T cells and tumor-associated macrophages (TAM) are recruited to the TME or acquire suppressive phenotypes in the TME [2]. While they are often immunosuppressive, TAM are highly plastic cells that can acquire a large spectrum of activation states and functions, depending on the stimuli from the environment. The simplistic classification of M1 and M2 macrophages with pro-tumor and anti-tumor functionality, respectively, represents the two extremes of the activation states that are typically used in *in vitro* cultures [6]. In the MM microenvironment, TAM are abundantly present and cytokines like IL-4, IL-10, and TGF-β promote their polarization toward a tumor-supporting phenotype [7–9]. This can shape TAM to secrete proinflammatory cytokines, which in turn further support MM progression [10, 11]. Additionally, TAM in MM are often described as M2-like and are amongst others a major source of the anti-inflammatory cytokine IL-10 [7, 10]. Macrophage polarization is thus multifaceted to support both tumor proliferation and an immunosuppressive milieu [2].

The immunosuppressive TME in MM can result in defective T cell- and NK cell-mediated tumor killing [12–14]. Hence, new therapy approaches aim to restore the dysfunctional antitumor responses, e.g., by blocking immune checkpoint (IC) molecules such as TIGIT or the PD-1/PD-L1 pathway and by adoptive transfer of modified T cells or NK cells [15]. IC are upregulated by inflammatory cytokines to prevent overactivation of the immune system, as well as by aberrant signaling pathways in tumor cells, including MM, to facilitate immune escape [16]. Although advances have been made, IC immunotherapies do not always achieve durable responses and, next to restoring antitumor functions of exhausted immune cells, reprograming of the TME toward a proinflammatory, and immunostimulatory TME may be crucial for sustained effectiveness of immunotherapies [17].

We and others focus on developing effective donor-derived NK cell-based therapy, which is considered a promising approach to restore impaired NK cell functionality in MM [18]. NK cells can rapidly and effectively kill tumor cells. To recognize their targets, NK cells express an array of activating and inhibitory receptors and attack when the activating signals outweigh the inhibitory ones [19]. Activating receptors for example bind to ligands upregulated in response to stress, while the major inhibitory receptors on NK cells are Killer-Immunoglobulin-like Receptors (KIRs) and NKG2A that bind to HLA class I molecules, which are expressed on all healthy cells. Next to natural cytotoxicity, the NK cells’ killing capacity can be enhanced through antibodies that induce antibody-dependent cellular cytotoxicity (ADCC) by binding to CD16, one of the most potent activating receptors on NK cells [20]. Additionally, NK cells can provide anti-tumor effects by promoting T helper 1 (Th1) immune responses through secretion of proinflammatory cytokines including high amounts of IFN-γ [21]. However, tumor cells have developed mechanisms to escape from NK cells, which can result in dysfunctional NK cell response in the MM environment. Therefore, different strategies have been exploited to create NK cells that remain functional in a suppressive TME. For tumor types like MM that are largely HLA class I^+^, donor NK cells can be used that possess KIRs for which the corresponding HLA ligands in the tumor are absent, resulting in a lower activation threshold and stronger NK cell anti-tumor cytotoxicity [22, 23]. ADCC is another way to induce robust NK cell effector functions. We have previously shown that ADCC-triggering by the clinically approved anti-CD38 antibody Daratumumab combined with KIR-ligand-mismatched NK cells helped to amplify the NK cell responses against MM tumor cell lines [22].

So far, NK cell therapies have been mainly assessed for their direct anti-tumor effects, i.e., killing tumor cells. In this study, we hypothesized that NK cells can further contribute to anti-tumor immunity through IFN-γ secretion by responding to myeloid-derived tumor-associated cells (TAC) and that such NK cell responses can be amplified by ADCC-triggering antibodies binding to TAC. We tested the response of IL-2-activated donor-derived NK cells in combination with the anti-PD-L1 blocking antibody Avelumab, known to trigger ADCC, on two types of in vitro macrophages that were either polarized toward a TAM-like phenotype or polarized toward a proinflammatory M1 phenotype. Moreover, we assessed the contribution of the inhibitory ligands HLA class I on TAM on NK cell functionality.

## Materials and methods

### Cell isolations and cell culture

Monocytes and NK cells were derived from buffy coats of healthy donors. The use of buffy coats does not require ethical approval in the Netherlands under the Dutch Code for Proper Secondary Use of Human Tissue. PBMCs were isolated by density gradient centrifugation with Lymphoprep (Axis Shield). Subsequently, monocytes were positively selected by CD14 MicroBeads (Miltenyi Biotec) and NK cells were isolated by negative selection using the NK cell isolation kit (Miltenyi Biotec) according to the manufacturer’s protocol. NK cells were cultured overnight at a density of 1.5 × 10^6^ cells/mL in RPMI1640 medium with Glutamax (Gibco), supplemented with 10% FCS and 1% P/S, plus 1000 U/mL recombinant human IL-2 (Proleukin, Novartis). If using fresh NK cells was not feasible, NK cells were frozen until further use and, prior to experiments, thawed and activated the same away as fresh NK cells. For the cytotoxicity assay, expanded NK cells were included: NK cells were expanded from CD3-depleted PBMCs in SGCM medium supplemented with 10% FCS, 1% Pen/Strep, and 1000 U/mL IL-2. After 17 days of expansion, NK cells were frozen and, prior to the experiment, thawed and recovered overnight in the presence of IL-2.

The multiple myeloma cell line L363 was used to polarize TAM. L363 was purchased from DSMZ Germany and cultured in RPMI1640 medium with Glutamax (Gibco) and supplemented with 15% fetal calf serum (FCS, TICO Europe) and 1% penicillin/streptomycin (P/S, Gibco). The cells were cultured in an incubator with conditions of 37°C, 21% O_2_ and 5% CO_2_.

### Monocyte polarization toward macrophages

CD14^+^ monocytes were plated in serum-free X-VIVO^™^ 15 medium (Lonza) with either 100 ng/mL recombinant human macrophage colony-stimulating factor (M-CSF, Immunotools) or 100 ng/mL recombinant human granulocyte–macrophage colony-stimulating factor (GM-CSF, Miltenyi Biotec) to differentiate the monocytes toward TAM or M1, respectively. On day 4, half-medium change was performed. On day 7, the cells were polarized toward TAM or M1 for 48 h as follows: To obtain TAM, 100 ng/mL M-CSF, 50 mM lactate (Sigma-Aldrich), 200 ng/mL prostaglandin E2 (PGE2; Sigma-Aldrich) were added together with either L363 cells in a 1:30 L363:monocytes ratio or with L363 supernatant. To obtain M1 macrophages, 100 ng/mL GM-CSF, 20 ng/mL recombinant human IFN-γ (rhIFN-γ, R&D Systems) and 10 ng/mL LPS (Sigma-Aldrich) were added to the medium.

To some conditions, supernatant from IL-2-activated NK cells or rhIFN-γ (R&D Systems) was added to the polarized macrophages for 24 h, referred to as NK supernatant. Additionally, 5 µg/mL LEAF purified anti-human CD119/IFN-γ R alpha chain, mouse IgG1 κ blocking antibody (clone GIR-208) or 5 μg/mL LEAF purified mouse IgG1 κ isotype control (MGI-45/ MOPC-21, both Biolegend) was added 30 min prior to adding the NK supernatant.

### Macrophage harvesting and flow cytometric analysis of macrophages

First, supernatant was removed and stored for CBA analysis, and then wells were carefully washed with PBS and 10 mM EDTA/PBS was added for 5 min at 37°C. Cells were subsequently gently harvested using a cell lifter. For the FACS staining, macrophages were blocked with the FcR blocking reagent, human (Miltenyi) prior to surface staining for CD80 APC-H7 (L307.4, BD), CD86 FITC (FM95, Miltenyi), HLA-ABC PE (REA230, Miltenyi), HLA-DR APC (AC122, Miltenyi), CD163 PerCPCy5.5 (GHI/61, BD), CD206 APC (19.2, BD), CD209 PerCPCy5.5 (DCN46, BD), and PD-L1 PE-Cy7 (REA1197, Biolegend) for 30 min in the dark and on ice. Directly before measuring the sample on the flow cytometer, 30 ng DAPI (Sigma-Aldrich) was added. To control for background staining, cells stained only with 30 ng DAPI were used. All flow cytometric analyses were performed with BD Canto II and data were analyzed with FlowJo 10.1r5 64-bit software.

### Cytokine profile via cytometric bead assay (CBA)

The stored supernatant was thawed prior to the assay and 50 μl of the supernatant per condition was prepared according to the protocol of the BD CBA Flex Set kit to measure the concentrations of IFN-γ, IL1-β, IL-6, IL-10, VEGF and TNFα (BD Biosciences).

### Cytotoxicity assay with L363 target cells

First, polarized macrophages were washed and co-cultured with IL-2-activated NK cells for 2 days. The NK cells were harvested by collecting the supernatant of the co-culture, while the macrophages adhered to the plates. Then, the macrophage-primed NK cells were co-cultured with the target cells L363, which were previously labeled with CellTracker^™^ CM-DiI Dye (Invitrogen) according to the manufacturer’s protocol. The co-culture was performed in 96-well plates with 2 × 10^4^ NK-cells and 2 × 10^4^ tumor cells per well (1:1 Effector:Target cell (E:T) ratio) for 4 h. After co-culture, the cells were stained with Live/Dead® Fixable Aqua Dead Cell Stain Kit (Invitrogen) for 30 min on ice and then fixed in PBS with 1% paraformaldehyde. The samples were measured on a BD CANTO II. Specific cytotoxicity was calculated as follows: $\% \,{\text{specific}}\,\,{\text{cytotoxicity}} = \frac{{(\% \,\,{\text{dead}}\,\,{\text{cells}} - {\text{average}}\,\% \,\,{\text{spontaneous}}\,\,{\text{death}})}}{{(100\% - {\text{average}}\,\% \,\,{\text{spontaneous}}\,\,{\text{death}})}}*100\%$

### CD107a degranulation assay, intracellular staining and IncuCyte analysis

Polarized macrophages were harvested and re-seeded in 96 well plates. Macrophages were incubated with 2 ug/mL Avelumab (MedChemExpress) or medium for 30 min before IL-2-activated NK cells were added in a 1:1 E:T ratio for 4-h co-culture. For the CD107a assays, CD107a-Horizon V450 antibody (H4A3, Miltenyi Biotec) was added immediately. After the first hour of co-culture, Monensin (BD Biosciences) was added and after another 3 h and the plates were put on ice to stop the assay. The cells were washed with PBS and first stained with Live/Dead® Fixable Aqua Dead Cell Stain Kit (Molecular Probes^™^) for 30 min on ice before surface staining with the following antibodies was performed for 30 min on ice: anti-CD3-APC-Vio770 (BW264/56), anti-CD56-PerCP-Vio700 (REA196), anti-KIR2DL1-APC (REA284), anti-KIR2DL2/3-PE (DX27), anti-KIR3DL1-FITC (DX9) and anti-NKG2A-PE-Vio770 (REA110). For IFN-γ staining, NK cells were subsequently treated with the Cytofix/ Cytoperm™ Fixation/Permeabilization Kit from BD according to the manufacturer’s protocol and stained with anti-IFN-γ (B27) for 30 min on ice. The assays were analyzed by flow cytometry with a BD CANTO II. For the image analysis using the Incucyte^®^ S3 Live-Cell Analysis System, macrophages were labeled with CellTracker^™^ CM-DiI Dye (ThermoFisher) and then co-cultured with NK cells as described above. Caspase-3/7 dye for apoptosis (Sartorius) was added according to the manufacturer’s procedure to detect cell death.

### Determination of KIR-ligand-matched and -mismatched NK cell subsets

The HLA class I genotype of the monocyte donors was determined by Luminex-SSO according to manufacturer’s protocol. Donors with an HLA-C1^+^ HLA-C2^+^ HLA-Bw4^+^ genotype were selected as NK cells donors for the assay to ensure that the analyzed KIRs are licensed. See Table 1 for determination of KIR-ligand-matched and -mismatched NK cell subsets.

**Table 1﻿:** KIR-ligand-matched and -mismatched NK cell subsets per macrophage donor

Macrophage donor 1, 4, 5	C1^+^	C2^−^	Bw4^−^
NK cell donors	KIR2DL2/3 = M	KIR2DL1 = MM	KIR3DL1 = MM

Macrophage donor 2	C1^−^	C2^+^	Bw4^+^
NK cell donors	KIR2DL2/3 = MM	KIR2DL1 = M	KIR3DL1 = M

Macrophage donor 3	C1^+^	C2^+^	Bw4^−^
NK cell donors	KIR2DL2/3 = M	KIR2DL1 = M	KIR3DL1 = MM

Macrophage donors were genotyped for HLA class I and classified as positive or negative for the HLA C1, C2 or Bw4 epitope groups. NK cells were derived from different donors, of which HLA class I genotype was determined to ensure that KIRs were licensed. NKG2A^−^ NK cell subsets were grouped into KIR-ligand-matched (M) or -mismatched (MM) subsets based on the HLA typing of the macrophage targets, e.g., for macrophage donor 1, 4, 5, the KIR2DL2/3 single-positive NK cell subset was considered matched, while KIR2DL1 single-positive, KIR3DL1 single-positive, and KIR2DL1^+^ KIR3DL1^+^ double-positive NK cell populations were considered mismatched

### Statistical analysis

All statistical analysis was performed with GraphPad Prism 9.2.0 (GraphPad Software Inc, San Diego, CA, USA) using nonparametric paired t-tests (Wilcoxon matched pairs tests). * indicates a *p*-value of < 0.05, ** indicates a p-value of < 0.01, and *** indicates a *p*-value of < 0.001.

## Results

### IL-2-activated NK cells degranulate and produce IFN-γ in response to in vitro polarized TAM and M1

**Fig. 1:**
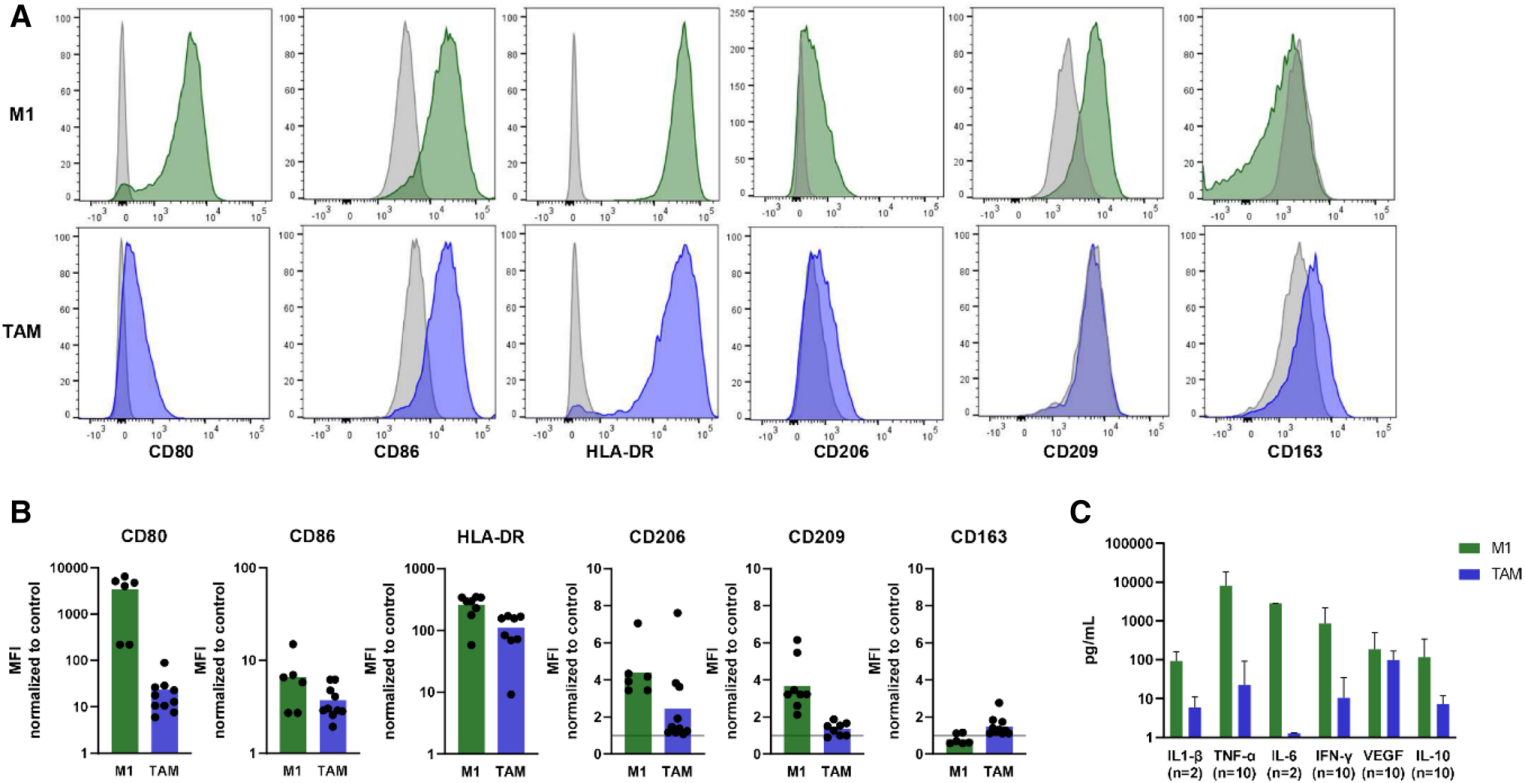
Phenotype of the macrophages polarized toward M1 and TAM. Monocyte-derived macrophages were polarized toward M1 or TAM phenotype. **A** After harvesting, the cells were stained for expression levels of the indicated markers and measured by flow cytometry. Representative histograms are shown. Grey histograms depict FMO, and green (M1) or blue (TAM) histograms depict staining with an antibody that is specific for the indicated marker. **B** Quantification of A. Expression on all donors depicted as normalized MFI (MFI of marker/MFI of FMO). Each dot represents one donor in monoplo and bars indicate the mean of all donors (*n* = 6–10). **C** Cytokine production was assessed in the supernatant of polarized M1 and TAM by CBA (*n* = 2 in duplo for all cytokines, additional *n* = 8 in monoplo for 4 cytokines). The values were corrected for the concentrations measured in the medium used for polarization. Bars indicate means + SD. TAM = tumor-associated macrophages, MFI = median fluorescence intensity, FMO = fluorescence-minus-one (unstained living cells)

To study NK cells responses against myeloid-derived TAC, we generated two types of macrophages, TAM and M1. TAM were generated by stimulating monocytes with M-CSF, PGE2, lactate as well as the MM cell line L363 to enable interaction of TAM with MM cells, as is expected to occur in the TME. Because the presence of L363 cells after harvesting TAM could influence NK cell responses, we also generated TAM with the same cocktail of M-CSF, PGE2 and lactate and with L363 supernatant instead of L363 cells (TAM-S). Additionally, we polarized macrophages toward the opposing end with GM-CSF, rhIFN-γ, and LPS to generate proinflammatory M1. Upon polarization, M1 displayed the typical long-stretched morphology, while TAM appeared spindle-like shaped and less stretched (Suppl. Fig. S1A). Since macrophages are highly versatile in their phenotype, we assessed the phenotypic profile of the generated macrophages by staining for cell surface markers and measuring cytokines in the supernatant of polarized macrophages. While we observed differences between donors regarding the expression levels of the different markers, all M1 expressed the co-stimulatory molecules CD80 and CD86 as well as high levels of the HLA class II molecule HLA-DR (Fig. 1A-B). Additionally, we assessed three receptors involved in host defense, of which CD206 and CD209 were expressed at low levels and CD163 was not expressed on M1 (Fig. 1A-B). The proinflammatory cytokines IL-1β, TNF-α, IL-6 and IFN-γ were produced by M1 as well as the vascular endothelial growth factor (VEGF) and IL-10 (Fig. 1C). Compared to M1, TAM expressed lower levels of the co-stimulatory molecule CD80, while HLA-DR was also present on TAM and the scavenger receptors CD206 and CD163 as well as the CD209 receptor were expressed at low levels (Fig. 1A-B). All assessed cytokines were measured in the TAM supernatant at low levels with secretion of VEGF being the highest of the measured cytokines (Fig. 1C). TAM-S displayed a comparable morphology and phenotype as TAM polarized with L363 cells (Suppl. Fig. S1A and S2).

**Fig. 2:**
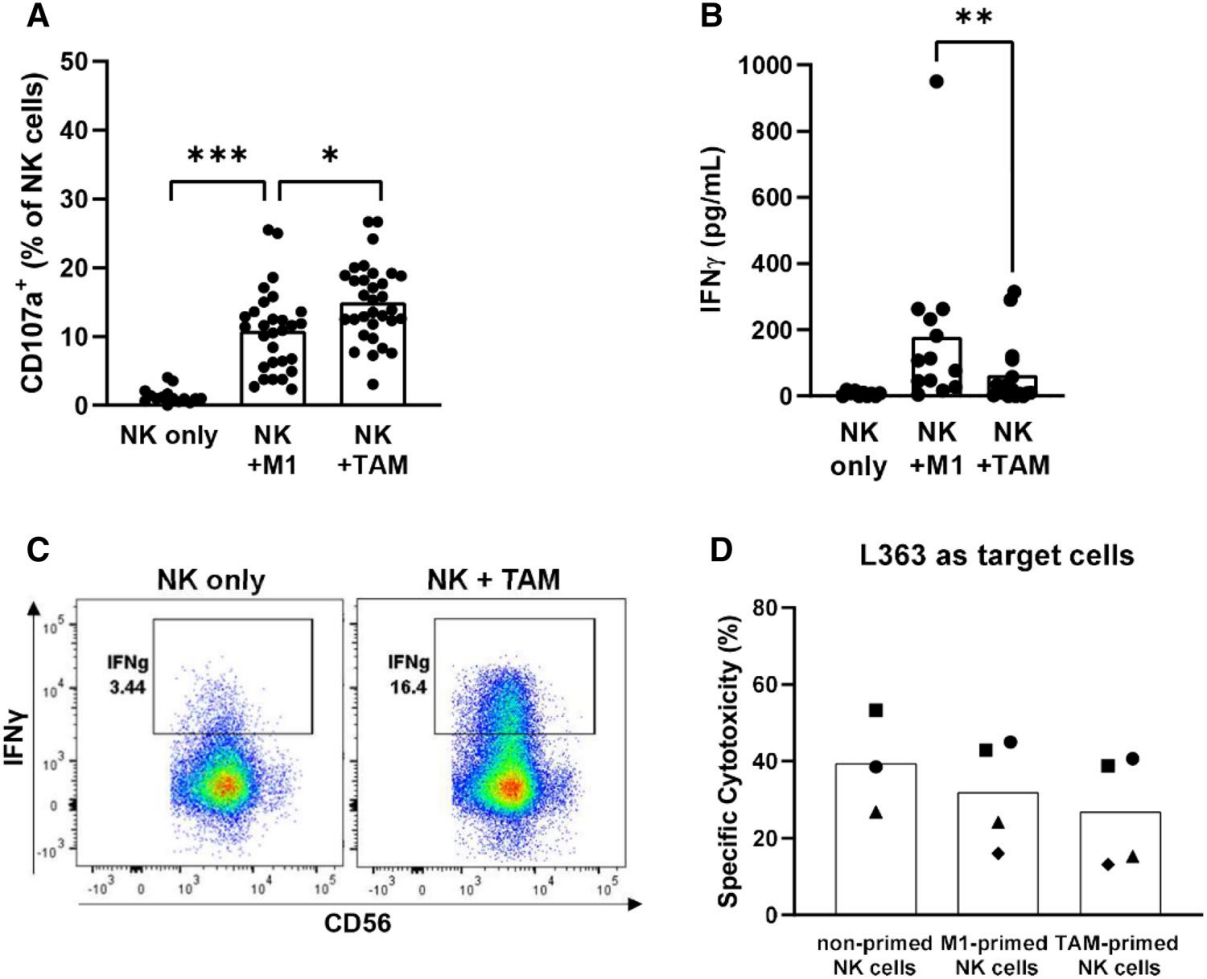
Cytokine-activated NK cells are triggered by in vitro-polarized macrophages. Monocyte-derived macrophages were polarized toward M1 or TAM phenotype and subsequently co-cultured with IL-2-activated NK cells. **A** Macrophages (8 donors) and NK cells (12 donors) in different combinations were co-cultured for 4 h, resulting in 29–32 unique macrophage-NK cell donor combinations depicted by the dots. NK cell degranulation (CD107a) against the macrophages as target cells was measured by flow cytometry in monoplo. **B** IFN-γ production in the supernatant upon 24 h co-culture of macrophages (4 donors) and NK cells (8 donors) was assessed by CBA in duplo. Dots show unique macrophage-NK cell donor combinations. **C** IFN-γ production by NK cells was determined by intracellular staining in the same setup as in B. Representative plots are shown and quantification is shown in Suppl. Fig. S3E. **D** Macrophages and NK cells were co-cultured for 24 h. The macrophage-primed NK cells were harvested and co-cultured with L363 target cells for 4 h in a 1:1 E:T ratio in duplo (cytotoxicity assay). Symbols represent the different NK cell donors. The percentage of dead L363 tumor cells is depicted as % specific cytotoxicity. In all graphs, bars represent means. Wilcoxon matched pairs tests were performed to determine statistical significance. Some of the donors in (**A,B**) are also used for Figs. 3 and 4

To test whether NK cell effector functions could be triggered or influenced by the interaction with macrophages, IL-2-activated donor NK cells were co-cultured with M1 or TAM and NK cell degranulation (CD107a) was assessed. After 4 h of co-culture, spontaneous degranulation of NK cells was below 5%, while up to 30% of NK cells degranulated in response to both M1 and TAM (mean of 11% against M1, 15% against TAM, Fig. 2A). Up to 20% of NK cell degranulation against TAM-S confirmed that NK cells responded to the macrophages (Suppl. Fig. S3A). To further study the effect of macrophages on the effector functions of NK cells, we determined IFN-γ levels in the supernatants after 24 h co-cultures of macrophages and NK cells. This revealed an average of 180 pg/mL and 40 pg/mL IFN-γ in the co-cultures with M1 and with TAM, respectively (Fig. 2B). Moreover, there was no obvious difference in the IFN-γ production in co-cultures with TAM or TAM-S (Suppl Fig. 3B). In supernatants from conditions with NK cells alone, TAM alone or M1 alone, IFN-γ was not produced, indicating that the IFN-γ secretion required the interaction between macrophages and NK cells (Suppl Fig S3C). While both macrophages and activated NK cells could produce IFN-γ, intracellular staining for IFN-γ confirmed that NK cells are producing IFN-γ in the co-cultures with macrophages (Fig. 2C and Suppl Fig. 3D-E). Despite NK cells responding to M1 and TAM, both types of macrophages did not seem to be killed by NK cells (Suppl Fig S3F).

Because TAM have been shown to inhibit cytotoxic effector functions of NK cells which would compromise the NK cell anti-tumor responses [24, 25], we also determined whether co-cultures with macrophages reduced the tumor killing capacity of activated NK cells in our setup. For this, M1 and TAM were co-cultured with IL-2-activated donor NK cells for 24 h. Afterward, NK cells were harvested and co-cultured with the multiple myeloma cell line L363 in a 4-h cytotoxicity assay. Without priming of macrophages, NK cells killed between 27 and 53% of L363 cells and this response was not strongly reduced when NK cells were primed by TAM or M1 (Fig. 2D). This illustrated that highly activated NK cells can be triggered to degranulate and produce IFN-γ by both macrophage types while their cytotoxic anti-tumor potential remained intact.

### NK cell degranulation and IFN-γ production in response to PD-L1^+^ M1 macrophage targets can be promoted with the ADCC-triggering antibody Avelumab

**Fig. 3:**
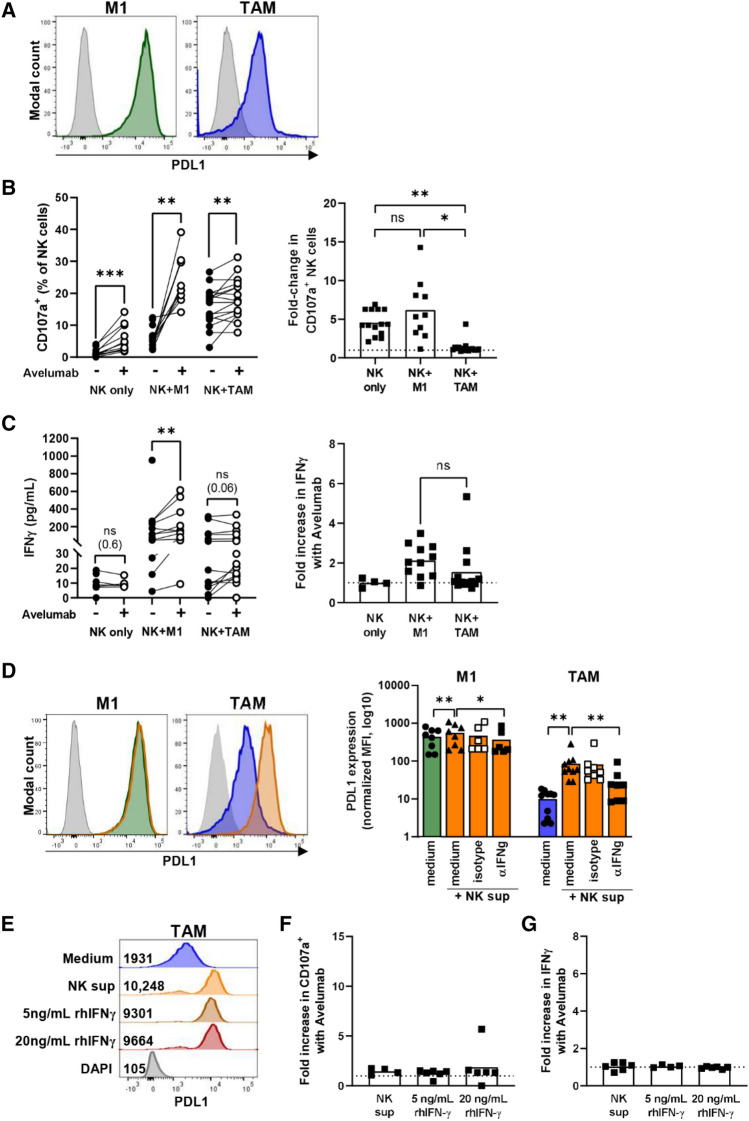
NK cell responses to PD-L1^+^ macrophage target cells can be promoted by Avelumab. **A** PD-L1 expression on M1 and TAM of a representative donor with the PD-L1 expression in green (M1), blue (TAM) and grey (FMO, unstained living cells). **B-C** Macrophages and activated NK cells were co-cultured for 4 h **(B)** or 24 h **(C)**. Macrophages (4 donors) were co-cultured with NK cells (8 donors) in different macrophage-NK cell donor combinations, resulting in up to 16 unique combinations (depicted as dots). The lines connect paired data points from the same donor with and without Avelumab. NK cell degranulation (CD107a) was measured by flow cytometry in monoplo **(B)**. IFN-γ levels were measured in the supernatants of the co-cultures by CBA in duplo **(C)**. **D** PD-L1 expression on M1 and TAMs after 24 h co-incubation with NK cell sup alone or in combination with IFN-γ receptor-blocking antibody or isotype control. Representative histograms are shown (orange = with NK cell sup) and quantification of PD-L1 expression is shown as normalized MFI (MFI of PD-L1-stained cells/MFI of FMO). Each dot represents one macrophage donor (*n* = 8–10 donors). **E–G** TAM (2 donors) were co-incubated with either NK sup or rhIFN-γ for 24 h. Subsequently, PD-L1 expression was assessed **(E)** and TAMs were co-cultured with NK cells (5 donors) for 4 h **(F)** or 24 h **(G)** and NK cell degranulation (CD107a, monoplo) and IFN-γ secretion (duplo) were assessed as described above. The data points without Avelumab are also included in Fig. 2. Fold increase with Avelumab compared to without Avelumab is depicted. Wilcoxon matched pairs tests were performed to determine statistical significance. NK sup = NK cell supernatant. rhIFN-γ = recombinant human IFN-γ

Since the production of IFN-γ could contribute to the overall anti-tumor effect of NK cells, we investigated whether the response could be enhanced by using clinically applicable ADCC-mediating antibodies that engage CD16, a powerful activating receptor on NK cells [20]. We observed that both M1 and TAM expressed PD-L1 and that M1 expressed PD-L1 at higher levels, most likely due to the polarization with rhIFN-γ (Fig. 3A). Because we detected PD-L1 expression on the macrophages, we used the anti-PD-L1 antibody Avelumab to enhance the NK cell response against macrophages. Importantly, Avelumab has been used in clinical studies and can, next to blocking interaction of PD-L1 and its ligand PD-1, also mediate ADCC [26]. To test NK cell degranulation against the macrophages with Avelumab, M1 or TAM were co-cultured for 4 h with NK cells in the presence of the anti-PD-L1 antibody Avelumab. IL-2-activated NK cells did not express PD-1 (Suppl. Fig. 4A). In the absence of macrophages, 0–4% of NK cells spontaneously degranulated without Avelumab and 1–14% of NK cells degranulated in presence of Avelumab (mean fold increase 4.5, Fig. 3B). Against M1, NK cells degranulation ranged from 2 to 12% without Avelumab (Fig. 3B). However, when Avelumab was added, degranulation against M1 was increased to 14–39% CD107a^+^ NK cells (mean fold increase 6.2, Fig. 3B). Against TAM, NK cell degranulation ranged from 3–26% and the addition of Avelumab resulted in a small increase in NK cell degranulation in 10 of 16 donors (mean fold increase 1.3, Fig. 3B). A comparable increase with Avelumab was also observed against TAM-S (Suppl Fig S4C).

To further assess the NK cell effector response, IFN-γ production was measured in the supernatant of 24 h NK cell–macrophage co-cultures. Avelumab itself did not enhance IFN-γ levels in cultures with only NK cells or only macrophages (Fig. 3C, Suppl. Fig. S4B). In line with the Avelumab-induced degranulation, more IFN-γ release was observed when Avelumab was added to M1 compared to conditions without Avelumab (Fig. 3C). Such a clear Avelumab-induced increase was not observed with TAM nor with TAM-S as target cells (Fig. 3C, Suppl Fig S4D).

Together, our results showed enhanced NK cell degranulation and IFN-γ production by Avelumab against M1 target cells with high PD-L1 expression and to some extent against TAM with lower PD-L1 expression.

### PD-L1 expression on macrophages was upregulated by NK cells in an IFN-γ dependent manner but did not further enhance the Avelumab-induced NK cell responses against TAM

Since IFN-γ, produced by activated immune cells, has been shown to enhance PD-L1 expression [27], we assessed whether cytokines produced by activated NK cells could enhance PD-L1 expression on macrophages and whether this could improve the effect of Avelumab on TAM. To study this, macrophages were exposed to NK supernatant derived from NK cells that were activated with a high dose of IL-2 for 24 h. No obvious changes in morphology were observed after treatment with NK supernatant (Suppl Fig S1B). PD-L1 expression was already high on M1 and was only slightly enhanced by addition of NK supernatant (1.3-fold on average), while PD-L1 expression on TAM was highly upregulated after incubation with NK supernatant (tenfold on average, Fig. 3D). The upregulation of PD-L1 on TAM was largely blocked by addition of an IFN-γ-receptor-blocking antibody, but not by an isotype control, demonstrating that the PD-L1 upregulation was largely mediated through IFN-γ (Fig. 3D). These results showed that highly activated NK cells produce IFN-γ in sufficient amounts to increase expression of the inhibitory checkpoint PD-L1 on macrophages. Additionally, we included conditions with two concentrations of rhIFN-γ to test if the PD-L1 expression on TAM could be enhanced further. Preincubation with both 5 ng/mL and 20 ng/mL rhIFN-γ enhanced PD-L1 expression to comparable levels as NK supernatant, which might suggest that the maximal expression under our in vitro culture conditions was reached (Fig. 3E).

Next, we tested whether the tenfold increase in PD-L1 on TAM after incubation with NK supernatant or rhIFN-γ coincided with stronger Avelumab-induced NK cell responses. This was not the case; the fold-increase in degranulation with Avelumab (compared to control without Avelumab) was less than twofold for TAM with NK supernatant and for TAM with rhIFN-γ (Fig. 3F), which was comparable to the fold-increase for TAM in medium (Fig. 3B). Similarly, Avelumab-induced IFN-γ release was not further enhanced by the higher expression of PD-L1 on TAM preincubated with either NK supernatant or rhIFN-γ (Fig. 3G). Against TAM-S, we observed the same responses as against TAM, supporting that the described responses are at least in part directed against the macrophages rather than against L363 (Suppl Fig. S4E-G).

Overall, our results indicate that increased PD-L1 expression on TAM after exposure to NK supernatant or rhIFN-γ did not further amplify the effect of Avelumab on NK cell responses against TAM.

### NK cell subgroups that do not encounter their HLA class I ligand degranulated stronger against macrophage target cells than NK cells that can interact with HLA class I

**Fig. 4:**
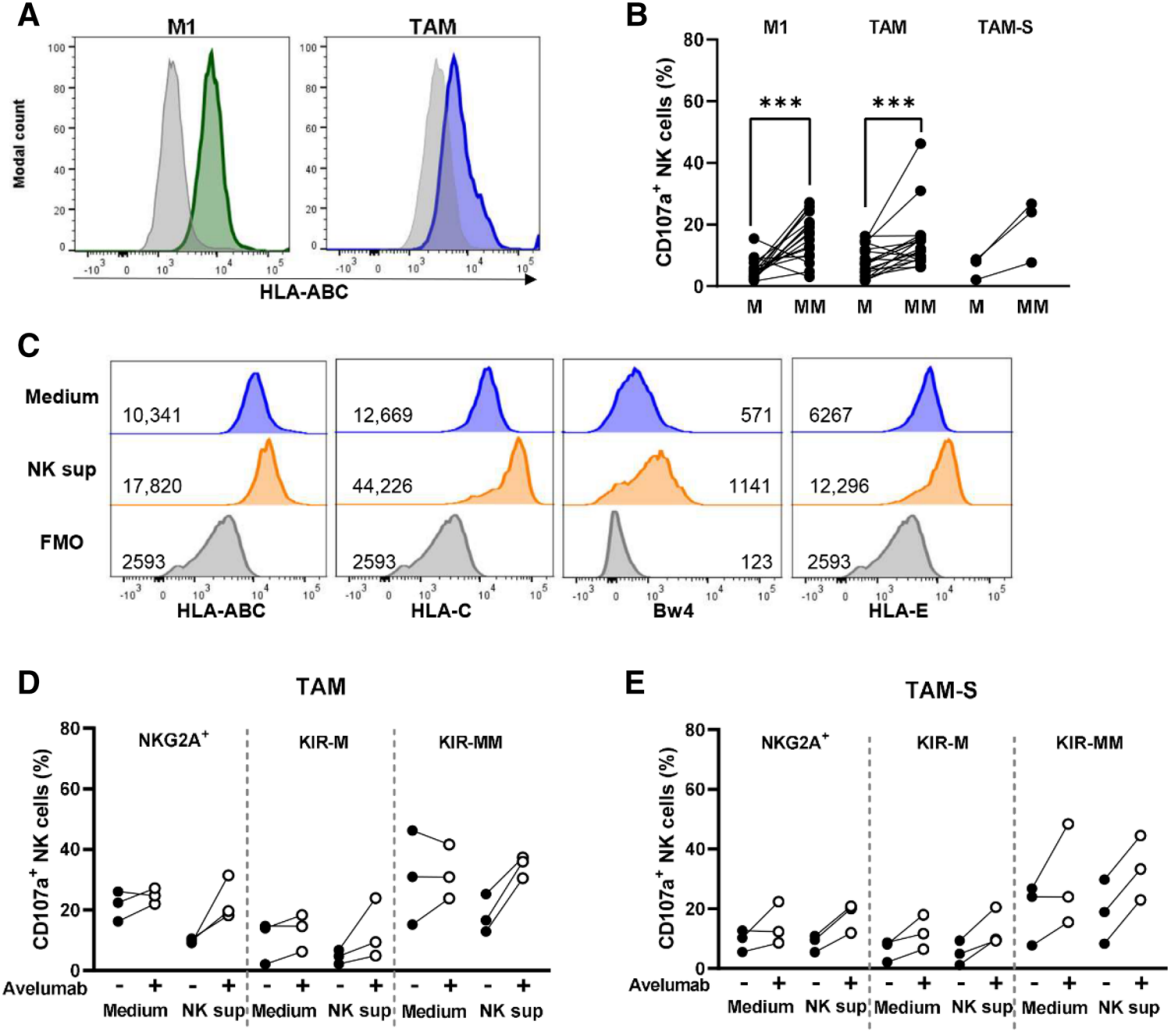
NK cell degranulation against macrophage targets was more pronounced in the KIR-ligand-mismatched NK cell subset than in the matched NK cell subset. **A** HLA-expression on M1 and TAM macrophages. **B-E** NK cell subpopulations were analyzed from the degranulation assay: CD56^+^ CD3^−^ NK cells were further divided in NK cell subgroups that were KIR-HLA ligand-matched (M) or -mismatched (MM). Degranulation of both subgroups is shown. **C** HLA expression on TAM in control medium or after 24 h incubation with NK sup. Living macrophages served as FMO. **D-E** NKG2A^+^ KIR^−^, NKG2A^−^ KIR-matched, NKG2A^−^ KIR-mismatched NK cell subsets were selected and their degranulation potential against TAM **D** or TAM-S **E** in medium vs. NK sup was compared with or without Avelumab (3 NK cell donors, 1 macrophage donor). Wilcoxon matched pairs tests were performed to determine statistical significance. NK sup = NK cell supernatant, FMO = fluorescent-minus-one

Since HLA class I is one of the most important inhibitors of NK cells, we studied whether HLA class I on macrophages is functionally relevant for NK cells. In our setup, both M1 and TAM expressed HLA class I (Fig. 4A). To investigate the relevance of HLA class I expression on macrophages, we analyzed the degranulation of NK cell subsets that encountered their cognate HLA ligand on macrophages (KIR-HLA ligand-matched NK cells) and NK cell subsets not encountering their ligand (KIR-HLA ligand-mismatched NK cells) in CD107a assays. Table 1 provides an overview of the KIR-ligand-matched and -mismatched NK cell populations.

Against M1 as target cells, the KIR-ligand-mismatched subgroup degranulated more vigorously than the matched subgroup in 16 of the 18 tested NK cell-target cell combinations (Fig. 4B). Against TAM, the difference in degranulation was less pronounced with some NK cell donors, but overall, the mismatched NK cell subsets degranulated more upon co-culture with TAM than the matched NK cells (12 of the 18 tested NK cell–target cell combinations) and this was also the case when TAM-S were used as target cells (Fig. 4B). The enhanced degranulation in the mismatched populations compared to the matched populations confirms that the HLA class I at least partly controls the magnitude of the NK cell response against macrophages.

HLA class I is known to be upregulated in response to IFN-γ [28]. To further study the functional relevance of HLA on the Avelumab-induced NK cell responses, we determined expression levels of all HLA class I on TAM upon incubation with NK supernatant. As expected, total HLA-class I as well as all the HLA molecules most relevant for NK cells (i.e., HLA-C, Bw4, and HLA-E) were upregulated on TAM after incubation with NK supernatant (Fig. 4C). HLA expression levels could potentially counteract or limit the ADCC-response of NK cells with Avelumab. To test this, we selected three NK cell subsets either exclusively expressing NKG2A, KIR-ligand-matched, or KIR-ligand-mismatched inhibitory receptors. The enhanced expression of HLA class I in response to NK supernatant did not result in an obvious difference in degranulation patterns between the three NK cell subsets (Fig. 4D-E). If enhanced HLA expression was limiting the Avelumab-induced response, it would be expected that the KIR-ligand-mismatched subset shows a stronger Avelumab effect than the other two subsets because the KIR-ligand-mismatched subsets lack interaction with their inhibitory HLA-C ligands. However, this was not the case and the increase in degranulation in combination with Avelumab was comparable for the subsets (Fig. 4D-E).

Our data therefore demonstrated that HLA class I are an inhibitor of NK cell responses against both M1 and TAM and that HLA expression at least partly mediates the magnitude of NK cell responses. Moreover, it suggests that enhanced HLA levels upon incubation with NK supernatant on TAM are not contributing to the absence of the Avelumab effect against TAM.

## Discussion

Creating a proinflammatory and immunostimulatory TME may be crucial for long-lasting effects of immunotherapies. In this study, we observed that IL-2-activated donor-derived NK cells degranulated and produced IFN-γ upon interaction with macrophages in our in vitro setup. The extent of NK cell reactivity against macrophages was, at least partly, controlled by HLA class I and could be further enhanced in combination with the ADCC-inducing antibody Avelumab against PD-L1-high M1 target cells, but not against PD-L1^+^ TAM. Importantly, we showed that the killing capacity of high-dose IL-2-activated NK cells remained comparable between macrophage-primed and unprimed NK cells. In previous studies, using a murine model or TAM derived from gastric cancer patients, NK cell responses such as degranulation and IFN-γ have been shown to be impaired [24, 29]. Our data provide evidence for the hypothesis that, even under suppressive conditions, activated NK cells can contribute to the overall anti-tumor immunity, on the one hand by direct cytotoxicity against tumor cells and on the other hand through production of IFN-γ which can enhance antigen presentation through HLA class I upregulation and stimulate Th1 and CD8^+^ T cell responses [21, 30]. This information is very valuable for strategies aiming to use adoptive transfer of donor NK cells that have been expanded and activated e*x vivo*.

Although NK cell-derived IFN-γ may promote type I immunity, IFN-γ is known to upregulate multiple factors including inhibitory molecules and might thus have opposing effects on immune cells including NK cells [31]. Moreover, IFN-γ can enhance expression of the immune checkpoint molecule PD-L1 on both tumor cells and other TME cells, hence, providing inhibition to tumor-infiltrating T cells with an exhausted PD-1^+^ phenotype [32]. To prevent this type of immune suppression, several clinically-approved antibodies are available to block the interaction between PD-1 and its ligand PD-L1. Of these antibodies, Avelumab is the only one that can also mediate ADCC by engaging the FcγRIIIa CD16 and thereby inducing strong NK cell responses against tumor cells [26]. In the present study, we showed that Avelumab-induced NK cell responses could also be directed against macrophages, most prominently against PD-L1-high M1. This demonstrates that the combination of NK cells and Avelumab may be also very relevant for tumors that lack expression of PD-L1 on tumor cells but that do contain PD-L1^+^ TAC in their TME.

In our study, both TAM and M1 expressed PD-L1, while Avelumab boosted degranulation and IFN-γ production of NK cells in response to M1 but not in response to TAM (Fig. 3). This may have to with the higher degranulation level against TAM in the absence of Avelumab, or with the slightly lower expression levels of the PD-L1 antigen on TAM vs M1. In previous studies, the relation between antigen expression levels and ADCC strength has been shown, for example, responses to PD-L1 blockade have been positively correlated with PD-L1 expression and a higher PD-L1 expression was found in breast cancer patients with complete response, compared to patients without complete response [33]; breast cancer cell lines with high PD-L1 expression were more sensitive to Avelumab-mediated ADCC [34]; and higher antigen expression and clustering on the cell surface have been correlated with stronger ADCC responses of Daratumumab (targeting CD38), Rituximab (targeting CD20), or Trastuzumab (targeting HER2) [22, 35–37].

We observed further upregulation of PD-L1 expression by NK-derived IFN-γ which was in line with a previous study showing that adoptively transferred expanded NK cells induced IFN-γ-mediated PD-L1 upregulation on tumor cells in xenograft models of lung cancer [38, 39]. In our study, the increased PD-L1 expression on TAM upon incubation with rhIFN-γ or NK cell supernatant did not translate in an increase in NK cell degranulation in the presence of Avelumab. In line with our data, a previous study evaluated PDL1 expression on a broad panel of cell lines and showed that IFN-γ increased PDL1 expression in all tested tumor cell lines. However, increased sensitivity to Avelumab-induced ADCC was observed only in some cell lines. Although no explanation for this effect was given, these data suggest that other factors may be influencing the overall result of Avelumab [26]. In our study, the lack of Avelumab effect could not fully be explained by an increase in expression of inhibitory HLA molecules upon incubation with rhIFN-γ or NK cell supernatant. As the level of expression matters, one explanation may be that the PD-L1 level on TAM was still not high enough to trigger potent activation of NK cells via Avelumab. In addition, IFN-γ could lead to the enhanced expression of additional factors or molecules that have been shown to affect NK cell activation positively, e.g., the adhesion molecules ICAM, or negatively, e.g., IDO [31, 40]. In a future study, it would therefore be interesting to generate a comprehensive overview of the differences in non-HLA ligands for NK cells expressed by M1 vs TAM and how they are influenced by IFN-γ or other factors secreted by activated NK cells as this may help to explain the differences between M1 and TAM that we observed.

The receptor PD-1 is frequently expressed on exhausted T cells, but in NK cells its role and functional relevance is still debated [41, 42]. While PD-1^+^ NK cells have been found in MM patients [43], several studies showed that cytokine-activated NK cells express no or only low levels of PD-1 [34, 41, 44]. In the situation where immune cells do express PD-1, Avelumab could thus have multiple effects: (1) Block the inhibitory effects of PD-1/PD-L1 interactions, which will release PD-1^+^ T- or NK cells from inhibition, (2) Mediate ADCC in NK cells against tumor or TME-associated PD-L1 expressing cells, and (3) Activate NK cells by engagement of CD16 via the Fc domains of the free floating Avelumab antibody, potentially improving anti-tumor activity in a manner that is not depending on formation of a lytic junction and cytotoxicity. In our current setup, we observed some effect of Avelumab on NK cell degranulation in the absence of target cells. As we did not observe PD-1 expression on NK cells in our study, it is unlikely that the Avelumab effect was mediated through the mechanism of blocking the PD-1/PD-L1 axis, although we cannot fully exclude it. Moreover, the combination of effects may be highly relevant when other ADCC-inducing antibodies are used that are directed against ligands of more abundantly expressed inhibitory receptors on NK cells.

In the current study we observed that KIR-ligand-mismatched NK cell subsets, for which the HLA ligand on macrophages was absent, degranulated stronger than the corresponding KIR-ligand-matched NK cell subsets, illustrating that HLA class I inhibited NK cell effector function. These results were in line with our previous findings with MM tumor cells, where we showed that selection of KIR-ligand-mismatched donors represents a strategy to lower the NK cell activation threshold [22]. The importance of HLA on macrophages has been highlighted in another study, where NK cells isolated from ovarian cancer patients could kill TAM that expressed low levels of HLA class I but not macrophages that expressed high levels of HLA class I [45]. In another study, activated NK cells have been shown to attack macrophages differentiated toward M0 and M2, while M1 macrophages were resistant to NK cells due to their higher expression of HLA class I [46]. In our study, M1 seemed to express higher levels of HLA class I than TAM. In agreement with this, the level of NK cell degranulation induced by M1 was slightly lower than the level of degranulation in response to TAM. Interestingly, IFN-γ production by NK cells seemed to be higher in the conditions with M1 than with TAM. This may be due to the difference in experimental setup and kinetics of both assays as degranulation was measured after 4 h of co-culture and IFN-γ after 24 h. It has been shown previously that NK cell degranulation and IFN-γ production do not always go in parallel [47]. Since NK cell activation is determined by the net balance of a broad array of receptors, ligands other than HLA class I or soluble factors could be complementary regulators of the NK cell responses, and M1 and TAM may differ in the expression of such non-HLA regulators.

One of the limitations of our study is the fact that we were not able to comprehensively quantify cell death of macrophages in our flow cytometry-based cytotoxicity assays because macrophages did not detach by pipetting and more harsh isolation procedures resulted in a high level of spontaneous macrophages death and unreliable analysis of NK-cell-mediated killing of macrophages. However, by evaluation of images, we observed that the macrophages in all conditions were still attached to the wells and did not undergo apoptosis, suggesting that both types of macrophages were not killed by the degranulating NK cells. Similar to lymphocytes, monocyte-derived cells can be equipped with protection mechanisms against misdirected granzyme B such as granzyme B antagonists, which could be one explanation for the absence of macrophage killing despite NK cell degranulation [48]. In addition, we determined the response of NK cells against macrophages as proxy for tumor accessory cells in general. We used macrophages as target cells because they are abundantly present and are generally considered a central mediator of tumor progression and poor prognosis in MM [49, 50]. In the TME, TAM can acquire profiles characterized by both pro-inflammatory and anti-inflammatory cytokines including IL-6 and IL-10 [11]. To mimic the interaction of TAM with MM cells, as it likely occurs in the TME, TAM in our study were generated with L363 cells or their supernatant. Tumor cell line-derived supernatant together with a cytokine-cocktail has been used previously to generate TAM in vitro [51]. A specific marker to identify TAM is not yet known due to high plasticity of TAM depending on received signals and heterogeneity in TME in MM. Commonly used TAM or M2-like markers include CD163 and CD206, also in MM [50, 52]. These markers are upregulated in response to stimulation with IL-10 and IL-4, which were not included in our cytokine cocktail and could thus be a reason why we observed rather low expression of these molecules on TAM in our study [53]. As expected, pro-inflammatory cytokines were expressed by M1 as was the anti-inflammatory cytokine IL-10, which could be a response to the LPS stimulation [54]. Compared to the other cytokines produced by TAM, we found high VEGF production, a factor known to be secreted by TAM in the MM BM and a main player of angiogenesis [52].

To include two ends of the macrophage spectrum, we generated M1 and TAM and the different NK cell responses suggest that the type of macrophage matters. In the TME of MM, the heterogeneity of TAM is, however, much more complex since they are exposed to the aberrant cytokine and signaling pathways of the TME [3]. Therefore, our study should be followed up in more complex models that better mimics the complex network in the TME of MM as well as the 3D tumor growth, for instance with in vivo models or tumor organoids that reflect TAM plasticity [55, 56]. In such models, it could also be assessed whether NK cell degranulation against M1 and TAM is continuous and could eventually lead to NK cell exhaustion. Moreover, it would be also very interesting to determine the response of NK cells against other cells residing in the MM TME, e.g., stromal cells, that can be PD-L1 positive and that may promote the cytokine-mediated antitumor effects of NK cells in combination with Avelumab.

Collectively, our data support the idea that, even in an immune-suppressive TME such as MM BM, highly activated NK cells could be triggered by TAC and could thereby serve as adjuvants of immune responses through the production of pro-inflammatory cytokines, such as IFN-γ. This would be an important addition to their direct anti-tumor function, i.e., killing tumor cells, and may contribute to improved long-lasting adaptive anti-tumor immunity.

### Supplementary Information

Below is the link to the electronic supplementary material.

Supplementary file1 (PDF 1028 KB)
