# Supplementary material for: Exploring the potential of combining IL-2-activated NK cells with an anti-PDL1 monoclonal antibody to target multiple myeloma-associated macrophages
Source: Cancer Immunol Immunother. 2023 Jan 19;72(6):1789–801. doi: 10.1007/s00262-022-03365-4 (PMC10198883; doi:10.1007/s00262-022-03365-4)
Supplement: Supplementary file 1 — Supplementary file1 (PDF 1028 KB) [file 262_2022_3365_MOESM1_ESM.pdf]

Supplementary Materials: Exploring the potential of combining IL-2 activated NK cells with an anti-PDL1 monoclonal antibody to target multiple myeloma-associated macrophages

Femke A. I. Ehlers, Niken M. Mahaweni, Annet van de Waterweg Berends, Thara Saya, Gerard M. J. Bos, and Lotte Wieten

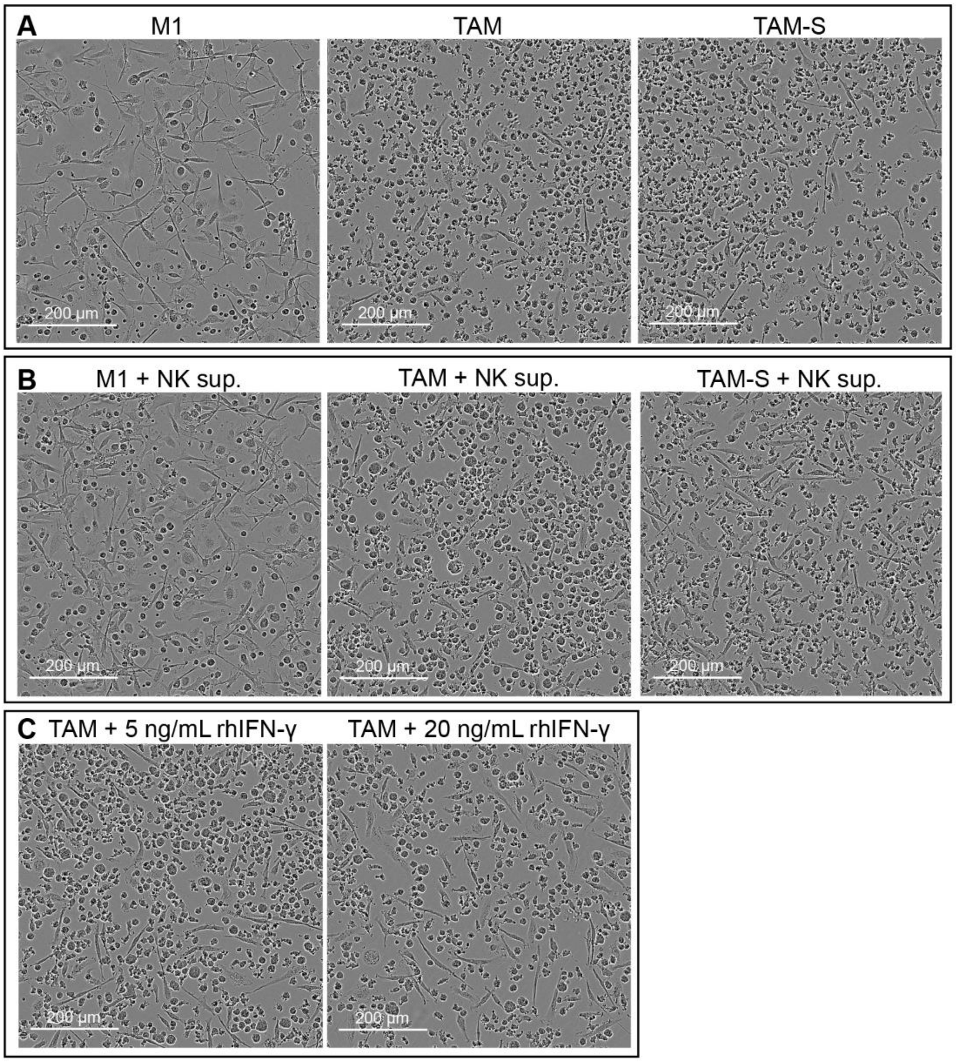

Supplementary figure S1. Morphology of macrophages polarized towards M1 or TAM. A-C) Macrophages were polarized towards M1, TAM or TAM-S and incubated in medium **(A)**, NK sup **(B)** or rhIFN-γ **(C)** for 24h. Conditions were performed in duplo. Images were taken with a IncuCyte ® S3 Live-Cell Analysis System before harvesting the cells. NK sup = NK cell supernatant, rhIFN-γ = recombinant human interferon-γ, TAM = TAMs generated with L363 cells, TAM-S = TAMs generated with L363 supernatant.

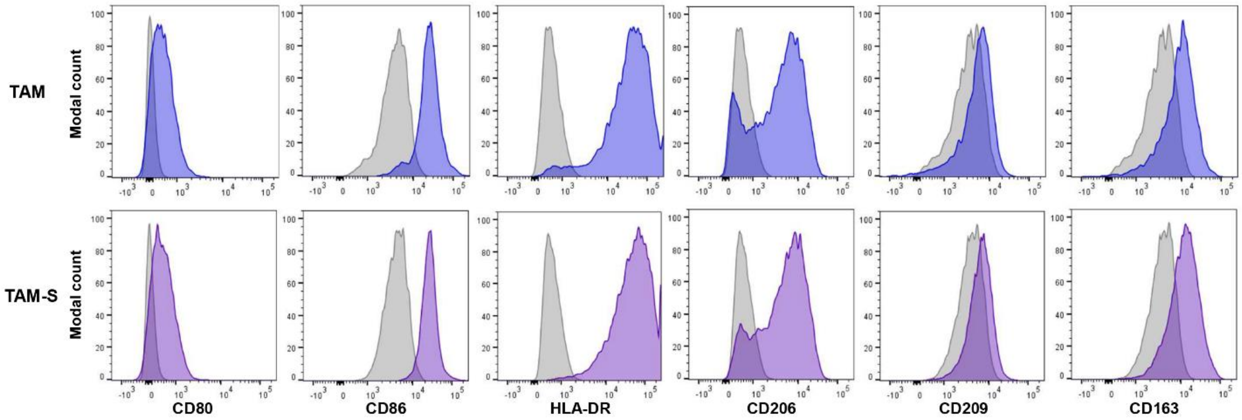

Supplementary figure S2. Phenotype of TAM polarized with L363 cells or with L363 supernatant. TAM-S were generated from 2 donors in monoplo and stained for the indicated cell surface markers and measured by flow cytometry. Grey histograms depict FMO and colored histograms depict staining with an antibody that was specific for the indicated marker. Histograms for TAM-S and TAM were generated from the same donor. TAM = TAMs generated with L363 cells, TAM-S = TAMs generated with L363 supernatant. FMO= Fluorescence-minus-one (unstained living cells).

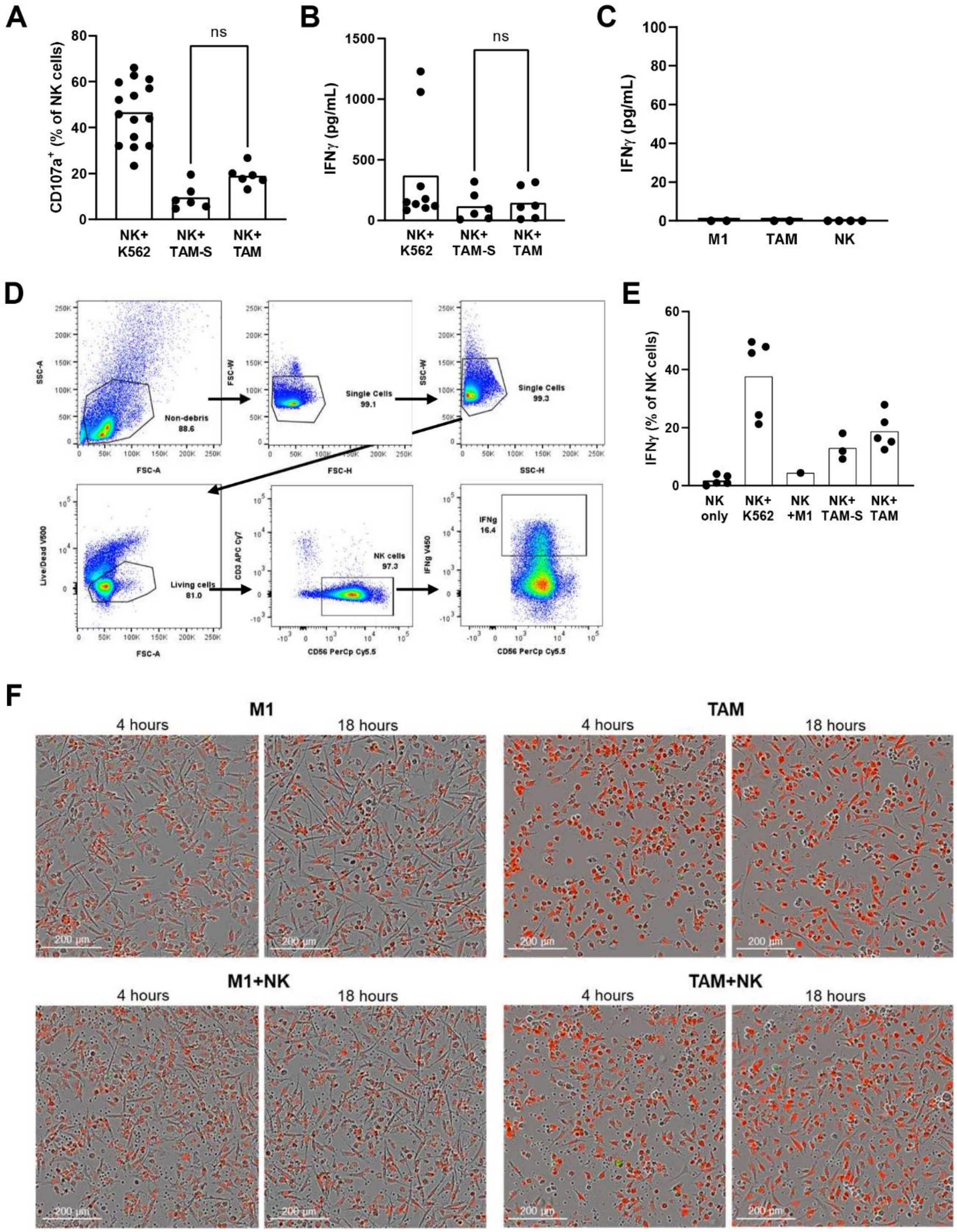

Supplementary figure S3. NK cell responses against the universal positive control cell line K562 and against TAM polarized with L363 supernatant. A) NK cell degranulation (CD107a) against the universal positive control cell line K562 or against TAMs that were polarized with L363 supernatant (TAM-S) in a 1:1 Effector:Target cell ratio. As comparison, corresponding data from TAM polarized with L363 cells, from Fig. 2A, is depicted. One dot = one NK cells donor, performed in monoplo. **B)** IFN-γ production was assessed in the supernatant of NK cells that were

cocultured for 24h with K562 or with TAM-S. IFN-γ was measured by CBA on a flow cytometer. One dot = one NK cells donor, performed in duplo. **C)** IFN-γ production as described in (B) in conditions with M1 alone, TAM alone or NK cells alone. **D)** Gating strategy to identify IFN-γ-producing NK cells by intracellular staining. **E)** Quantification of IFN-γ production by NK cells (5 donors total) against macrophages (2 donors) detected by intracellular staining. **F)** M1 and TAM were labeled with CM-DiI (red fluorescence), seeded and co-cultured with NK cells (unlabeled) in the same conditions as for CD107a experiments. Caspase-Glo 3/7 (green fluorescent) was added to all conditions. Images were taken with a IncuCyte® S3 Live-Cell Analysis System at the indicated timepoints. Wilcoxon matched pairs tests were performed to determine statistical significance in (A) and (B).

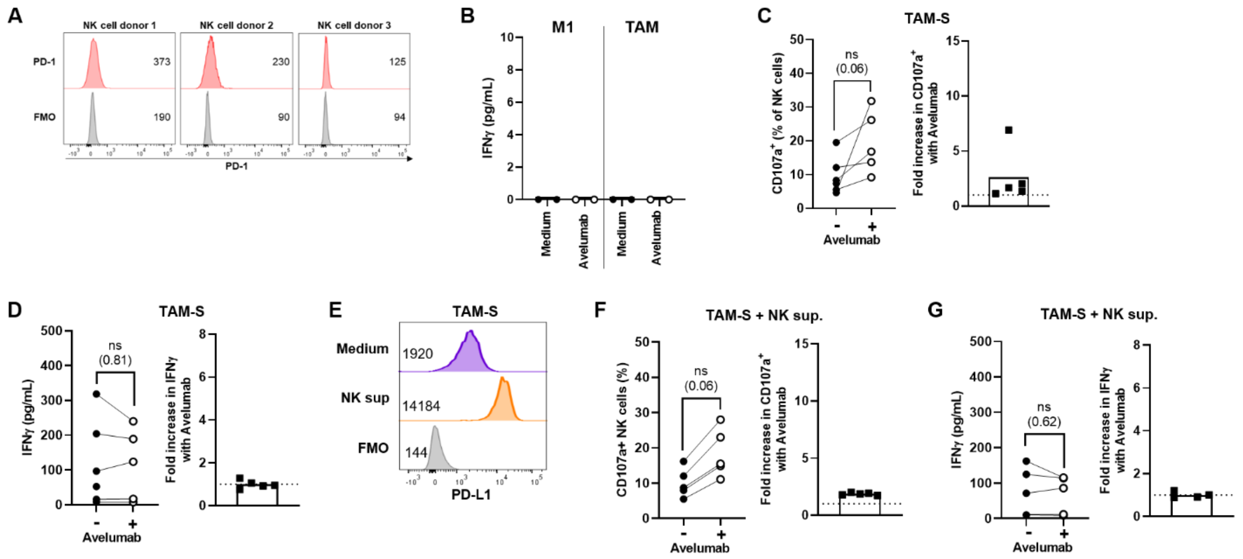

Supplementary figure S4. NK cell responses in combination with Avelumab. (A) PD-1 expression on IL-2 activated NK cells, derived from 3 donors. **(B)** IFN-γ levels detected in the cultures with macrophages only, measured in duplo. **(C-G)** NK cell responses against TAM-S: **(B, F)** NK cell degranulation (CD107a) against TAM-S after 4h co-culture, performed in monoplo. **(C, G)** IFN-γ production after 24h co-cultures measured by CBA in duplo. E**)** PD-L1 expression on TAM-S after culture in medium alone or after NK sup (in monoplo), with FMO depicted in grey. In all graph with lines, the lines connect paired data points of one macrophage-NK donor combination with and without Avelumab. Bars depict means of all donors.
